# Frequency Modulation Approach for High Power Density 100 Hz Piezoelectric Vibration Energy Harvester

**DOI:** 10.3390/s22239493

**Published:** 2022-12-05

**Authors:** Dengfeng Ju, Lu Wang, Chunlong Li, Hui Huang, Hongjing Liu, Kewen Liu, Qian Wang, Xiangguang Han, Libo Zhao, Ryutaro Maeda

**Affiliations:** 1 State Grid Smart Grid Research Institute Co., Ltd., Beijing 102209, China; 2 Electric Power Intelligent Sensing Technology and Application State Grid Corporation Joint Laboratory, Beijing 102209, China; 3 State Key Laboratory for Manufacturing Systems Engineering, International Joint Laboratory for Micro/Nano Manufacturing and Measurement Technologies, Xi’an Jiaotong University (Yantai) Research Institute for Intelligent Sensing Technology and System, Xi’an Jiaotong University, Xi’an 710049, China; 4 School of Mechanical Engineering, Xi’an Jiaotong University, Xi’an 710049, China; 5 Shandong Laboratory of Yantai Advanced Materials and Green Manufacturing, Yantai 265503, China; 6 State Grid Beijing Electric Power Research Institute, Beijing 100075, China; 7 Standard Verification Laboratory for On-Site Testing Technology, Beijing 102209, China

**Keywords:** resonant frequency, vibration energy harvesting, frequency modulation, magnetic coupling

## Abstract

Piezoelectric vibration energy harvester (PVEH) is a promising device for sustainable power supply of wireless sensor nodes (WSNs). PVEH is resonant and generates power under constant frequency vibration excitation of mechanical equipment. However, it cannot output high power through off-resonance if it has frequency offset in manufacturing, assembly and use. To address this issue, this paper designs and optimizes a PVEH to harvest power specifically from grid transformer vibration at 100 Hz with high power density of 5.28 μWmm^−3^g^−2^. Some resonant frequency modulation methods of PVEH are discussed by theoretical analysis and experiment, such as load impedance, additional mass, glue filling, axial and transverse magnetic force frequency modulation. Finally, efficient energy harvesting of 6.1 V output in 0.0226 g acceleration is tested in grid transformer reactor field application. This research has practical value for the design and optimization process of tunable PVEH for a specific vibration source.

## 1. Introduction

The sustainable state monitoring of power grid transformers by wireless sensor nodes (WSNs) is important. Only battery power supply for WSN works for a short time and is not easy to replace. Piezoelectric vibration energy harvester (PVEH) has been widely investigated as a promising device for sustainable power supply of WSN [1]. For example, vibration energy harvesting with permanent magnet [2] caused by magnetic field excitation can be used in self-powered alternating current (AC) power line monitoring devices in smart grid applications [3]. In addition to the magnetic field excitation, the mechanical vibration excitation of the shell is also widespread. The base vibration frequency of transformers is 100 Hz, but the amplitude is generally low, near 0.1 g [4]. To ensure the milliwatt level power supply of WSN under such low acceleration excitation, it requires the PVEH to have high power density at the accurate resonant frequency [5].

Despite being micro electromechanical systems (MEMS), PVEH has near 100 Hz resonant frequency; the output power is relatively low at 0.1 g acceleration [6] because the resonator mass is light and has low electromechanical coupling coefficient due to a thin piezoelectric layer [7]. Using the high electromechanical coupling piezoelectric materials such as PZT, reducing structural damping and increasing the mass block [8] are three key points to achieve high power output PVEH. Piezoelectric cantilever beam with large rectangular [9] shaped or L shaped [10] tungsten proof mass is often used in PVEH design to increase the vibration energy. Because most PVEH test prototypes [11] use the bolt clamped [12] piezoelectric cantilever beam [13], the damping is difficult to reduce, and the resonant frequency is easy to drift and there are gaps. Therefore, the bonding and glue filling assembly process is more suitable for PVEH product. Vacuum packaging also contributes to decrease mechanical damping ratio [14], but vacuum packaging also has leakage problems.

Linear PVEH resonant frequency needs to be strictly aligned with the excitation frequency to maximize harvested power. In the design phase, the PVEH structure is optimized, and resonant frequency is determined by theoretical or finite element simulation [15]. PVEH resonant frequency might vary in the manufacturing phase because of machining and assembly errors. It also might change in field assembly, or vary during operation with time because of environmental variations, leading to harvested power decrease. Although many nonlinear technologies have been proposed to broaden resonant frequency, linear PVEH is suitable for harvesting energy from small excitation with constant frequency of equipment such as a power grid transformer. Therefore, it is meaningful to study the linear PVEH resonant frequency modulation method.

PVEH resonant frequency is dependent on mass and stiffness. It is easier to change the stiffness than mass by indirect ways, such as applying mechanical force [16], magnetic force [17] or piezoelectric force [18] on the PVEH to modulate the resonant frequency. It is a trend to replace manual regulation with automatic regulation. Piezoelectric force can be adjusted by electrical load impedance, and the stronger the electromechanical coupling system, the stronger the piezoelectric force. Piezoelectric force type also has low power consumption when controlling electrical load impedance by an adaptive switch [19].

The frequency modulation methods of mechanical force or magnetic force [20] have been proved to be effective in the prototype with strong excitation and large amplitude [21], but whether they are applicable in the case of weak excitation and small amplitude is questionable. The frequency modulation effect by piezoelectric force is dependent on the electromechanical coupling strength [22].

This paper gives the design method of 100 Hz resonant PVEH structure in COMSOL simulation and proposes 3D printing aluminum alloy frame gluing packaging process to ensure low damping and high power density. This paper discusses the modulation methods of PVEH resonant frequency deviation by theoretical analysis and experiment, such as load impedance, additional mass, glue filling, axial and transverse magnetic force frequency modulation. A field application in grid transformer reactor is implemented to prove the value of designed tunable PVEH.

## 2. Structure and Mechanism

### 2.1. PVEH Structure and Working Principle

**Figure 1:**
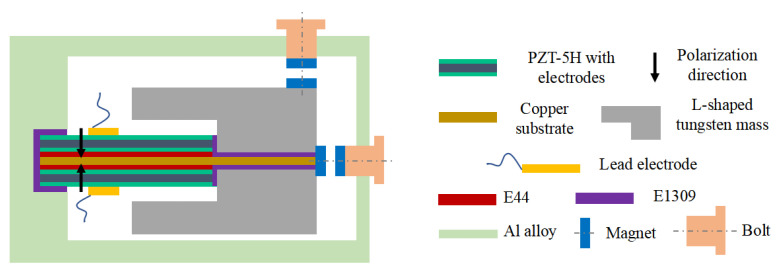
Tunable PVEH structure diagram.

The tunable PVEHs designed in this paper are composed of piezoelectric bimorph, L-shaped tungsten mass and aluminum alloy frame, as shown in Figure 1. The piezoelectric bimorph is composed of two PZT-5H sheets and the copper substrate layer in the middle by E44 epoxy resin bonding. PZT-5H sheets are covered with Ag electrodes and form series connection with opposite polarization direction. The L-shaped tungsten mass is used to ensure high power density. Aluminum alloy frame is fabricated by 3D printing. Assembly processes use E1309 AB glue bonding in mass, bimorph and frame clamping. The M5 bolt bonding with NdFeB magnet inserts into the frame to adjust the resonant frequency.

### 2.2. Modeling and Simulation

**Figure 2:**
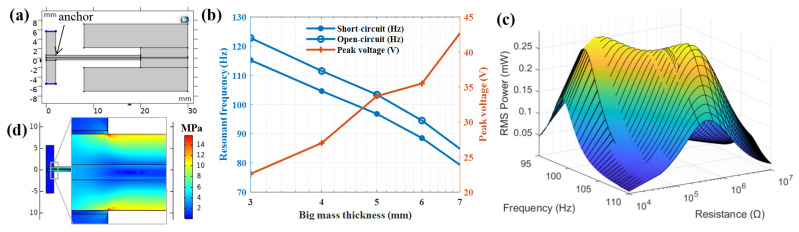
PVEH modeling and simulation: (**a**) PVEH 2D model; (**b**) PVEH resonant frequency and peak voltage varied with big mass thickness; (**c**) RMS power in different load resistance near resonant frequency of PVEH with 5 mm big mass thickness; (**d**) stress check cloud chart.

**Figure 3:**
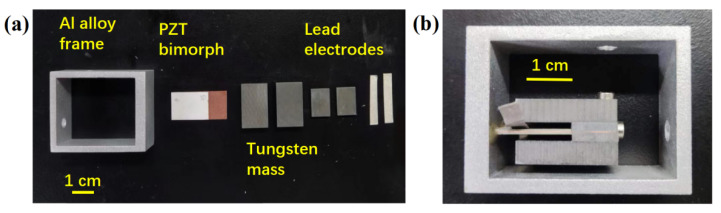
(**a**) PVEH assembly parts and (**b**) PVEH prototype.

Finite element analysis (FEA) is used in the modeling and simulation analysis of PVEH in COMSOL Multiphysics as shown in Figure 2a. A 2-dimensional model of PVEH is established for eigenfrequency, stress check and electromechanical coupling frequency response analysis in 0.1 g acceleration. Design method of L shape mass is available in reference [10]. The big mass thickness is optimized in PVEH design. PVEH resonant frequency decreases in both open circuit (10 MΩ) and short circuit (1 kΩ) state, and open-circuit peak voltage increases with the increase in big mass thickness as shown in Figure 2b. To match the grid transformer vibration of 100 Hz, the big mass thickness is chosen as 5 mm. RMS power in different load resistance near resonant frequency of PVEH with 5 mm big mass thickness is plotted in Figure 2c. Two peak power points near 0.3 mW are found in 96.8 Hz at 600 kΩ and 103.4 Hz at 50 kΩ. A flat saddle area between the two peak power points proves a certain broadband energy harvesting ability. Half peak power of 0.15 mW can be reached between 95 Hz to 110 Hz. The stress check cloud chart in Figure 2d shows maximum stress of 15 MPa at the anchor of the cantilever, which is less than PZT allowable stress. Finally, PVEH parameters are designed and optimized, as shown in Table 1. PVEH assembly parts and prototype are shown in Figure 3.

## 3. Results and Discussion

### 3.1. Load Impedance Frequency Modulation

**Figure 4:**
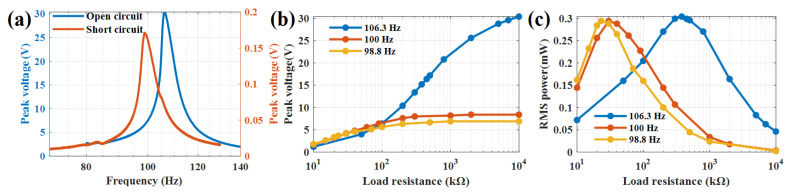
PVEH frequency modulation result by load impedance: (**a**) PVEH experimental peak voltage frequency response curves at open circuit (load resistance of 10 MΩ) and short circuit (load resistance of 1 kΩ); PVEH experimental (**b**) peak voltage and (**c**) RMS power in different load resistance at 106.3 Hz, 100 Hz and 98.8 Hz, respectively.

PVEH prototype performance is tested in a vibration platform (Econ VT9008) and exciter (JZK-50). PVEH was given 0.1g acceleration excitation and the frequency was swept from 60–140 Hz and the frequency response curve in open circuit (10 MΩ) and short circuit (1 kΩ) state was identified. Experimental results show the resonant frequency in open circuit and short circuit state are 106.3 and 98.8 Hz, respectively. At the two resonant frequency points and 100 Hz, the voltage and power on the load resistance are tested and the results are shown in Figure 4.

It can be seen that the open circuit voltage at 106.3 Hz, 100 Hz and 98.8 Hz is 30.4 V, 8.4 V and 6.9 V, and the maximum power is 0.304 mW, 0.294 mW and 0.2944 mW at the optimal load of 380 kΩ, 30 kΩ and 22 kΩ, respectively. The output power at 100 Hz has peak value as long as there is 100 Hz between the resonant frequency in open circuit and short circuit state.

PVEH system parameter identification results are shown in Table 2. The bimorph anchoring process by filling glue and inserting into aluminum alloy frame ensures the high mechanical quality factor. Tight bonding of bulk PZT ensures the highly effective electromechanical coupling coefficient, which ensures the high figure of merit *FoM* = *k_e_*^2^*Q*. When the *FoM* is bigger than 2, the system is strongly coupling [23] and behaving as broadband peak power by changing the load impedance.

### 3.2. Proof Mass Frequency Modulation

**Figure 5:**
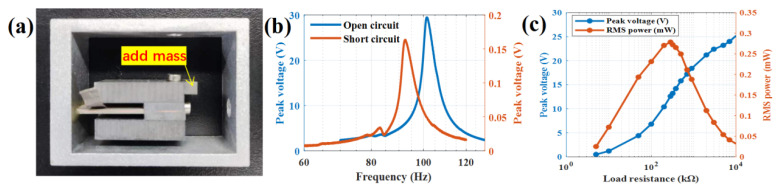
PVEH frequency modulation result by adding mass: (**a**) PVEH prototype with added mass; (**b**) PVEH experimental peak voltage frequency response curves at open-circuit (load resistance of 10 MΩ) and short-circuit (load resistance of 1 kΩ); (**c**) PVEH experimental RMS power in different load resistance at 100 Hz.

If the tested short circuit resonant frequency of the PVEH prototype is beyond the aim frequency of 100 Hz due to fabrication error, it can be reduced by adding the proof mass approach. Putting the added mass on the left end of the L shape mass will not impact sensitivity. Putting the added mass on the top end of the L shape mass will impact stopper spacing. Putting the added mass on the right end of the L shape mass is most sensitive and effective. Here, the PVEH prototype in the previous section is still used by gluing pieces of tungsten on the right end of the existing L-shaped proof mass. The added mass of 3.8 g PVEH prototype and experimental results are shown in Figure 5.

Experimental results shown the open circuit resonant frequency of PVEH is reduced from 106.3 Hz to 101.4 Hz, and the short circuit resonant frequency is reduced from 98.8 Hz to 92.6 Hz after the end mass is added, which effectively reduces the resonant frequency of PVEH. The output power of PVEH at 100 Hz is 0.2785 mW, which is slightly lower than that before frequency modulation, because the quality factor decreases.

The frequency modulation by adding proof mass only decreases the resonant frequency of PVEH. Indeed, removing some proof mass can increase the resonant frequency of PVEH, but it is not easy to handle. In some of the literature [24], it is feasible to adjust the resonant frequency by rotating the bolt and changing the position of the center of mass. Another way to increase the resonant frequency of PVEH is adding more glue on the anchor. In our experiment, resonant frequency of PVEH is increased about 5 Hz in this way. However, it is difficult to quantify the amount of glue applied and the effect of frequency rise.

To investigate whether gravity direction affects the PVEH resonant frequency, the PVEH prototype is set horizontally and vertically along the gravity direction, and the deviation result is less than 0.1 Hz in the test. The equation for tuning a cantilever beam by axial force can explain it.

(1) $$f_{r1}=f_{r0}\sqrt{\left(1-\frac{P}{P_{cr}}\right)},\;P_{cr}=\pi^{2}EI/4L^{2}$$

where the *f*_*r*0_ and *f*_*r*1_ are the original and adjusted resonant frequencies, respectively. *P* and *P_cr_* are the axial load and Euler critical buckling load of the cantilever beam, respectively. For the cantilever beam, *E* is the elastic modulus, *I* is the moment of inertia of the section, and *L* is the beam length.

The short and thick cantilever beam has *P_cr_* of 542 N, and the gravity of the vibrator is only 0.65 N, which has deviation of 0.06 Hz in theoretical calculation. To increase the influence of axial force, the magnetic force is used in the next section.

### 3.3. Magnetic Frequency Modulation

Field assembly also has an impact on the resonant frequency of PVEH. To adjust the PVEH resonant frequency when mounted on the vibrational device, such as a grid transformer, the method of magnetic frequency modulation is introduced, and the system stiffness is changed by magnetic force for frequency modulation. This paper compares the effect of axial magnetic force and the transverse magnetic force frequency modulation method.

**Figure 6:**
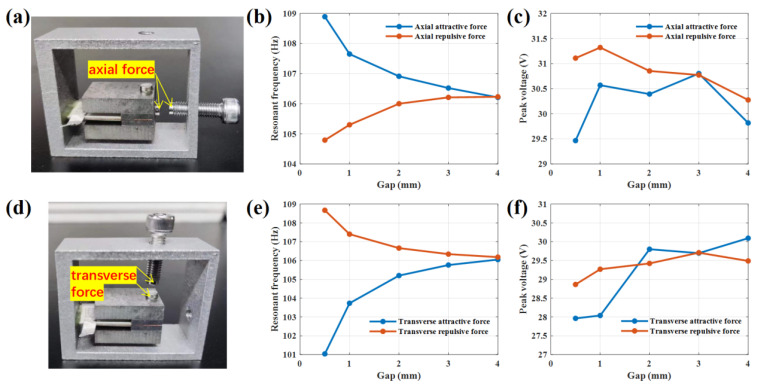
PVEH frequency modulation method and result by magnetic force: (**a**) PVEH prototype with axial magnetic force; (**b**) Resonant frequency and (**c**) voltage at open-circuit (load resistance of 10 MΩ) by axial attractive force and repulsive force with a different magnetic gap; (**d**) PVEH prototype with transverse magnetic force; (**e**) Resonant frequency and (**f**) voltage at open circuit (load resistance of 10 MΩ) by transverse attractive force and repulsive force with different magnetic gap.

The magnetic frequency modulation PVEH prototype is shown in Figure 6; here, (a) is the axial one, and (d) is the transverse one. The stiffness of the PVEH vibration system is changed by applying magnetic force. Two circular magnetic blocks with a diameter of 4 mm and a thickness of 2 mm are, respectively, fixed at the center of the free end of PVEH and the end of an adjustable bolt. The gap between the two circular magnetic blocks is controlled by the adjustable bolt to control the size of the magnetic force. Change is from repulsion to attraction by replacing the polarity of the magnet, and the stiffness of PVEH vibration system is increased/decreased by controlling the size of repulsion/attraction to achieve the purpose of increasing and reducing the resonant frequency.

The magnetic block spacing was controlled at 0.5 mm, 1 mm, 2 mm, 3 mm, 4 mm and 5 mm, respectively, and then the frequency reduction effect of repulsion, and the frequency increase effect of attraction, and their impact on the output characteristics of PVEH was explored.

Experimental results of resonant frequency and voltage at open circuit (load resistance of 10 MΩ) by axial attractive force and repulsive force with different magnetic gaps are shown in Figure 6b and Figure 6c, respectively. When the magnetic block gap is 4 mm, no matter whether the magnetic block is attractive or repulsive, there is no obvious frequency modulation effect. As the magnetic block spacing gradually decreases to 0.5mm, the magnetic force between the two magnetic blocks gradually increases. When PVEH is axial repulsed, its resonant frequency decreases from 106.3 Hz to 104.8 Hz, and the frequency modulation range is 1.5 Hz; when PVEH is axial attracted, its resonant frequency increases from 106.3 Hz to 108.9 Hz, and the frequency modulation range is 2.3 Hz. In magnetic frequency modulation, the axial magnetic force has little effect on the open circuit voltage of PVEH, which is always in the range of 29.5–31.5 V.

The transverse magnetic force frequency modulation is equivalent to a spring. But the frequency modulation effect is opposite to the axial force frequency modulation effect, as shown in Figure 6e,f, respectively. When PVEH is transverse repulsed, its resonant frequency increases from 106.3 Hz to 108.7 Hz, and the frequency modulation range is 2.1 Hz; when PVEH is transverse attracted, its resonant frequency decreases from 106.3 Hz to 101 Hz, and the frequency modulation range is 5.3 Hz. Although its frequency increase effect is almost the same as that of axial force, its frequency decrease effect is significantly better than that of axial force. In transverse magnetic frequency modulation, the magnetic force also has little effect on the open circuit voltage of PVEH, and the open circuit voltage is always in the range of 28–30.5 V.

A larger magnet has more force to achieve a larger tuning bandwidth. However, the space is limited, as is the magnet force. Many studies have demonstrated the wide frequency modulation capability of magnetic coupling, due to their low resonant frequency and low stiffness. For our 100 Hz PVEH having rigid stiffness, the *P_cr_* is high, and axial force has weak influence on resonant frequency.

### 3.4. Performance Comparison

The performance comparison of various PVEHs is shown in Table 3. Here, active volume is defined as the cubic volume occupied by piezoelectric beam and proof mass. Our PVEH has a considerable normalized power density (NPD) of 5.28 μW mm^−3^ g^−2^ with active volume of 5566 mm^3^ (28 mm × 14 mm × 14.2 mm). Mechanical quality factor is a key factor to infect NPD. The quality factor of rigid clamping by glue filled packaging in this work is higher than that of flexible clamping by electrode in our previous work [10]. The quality factor will be improved in our future work by more optimization.

### 3.5. Field Application

**Figure 7:**
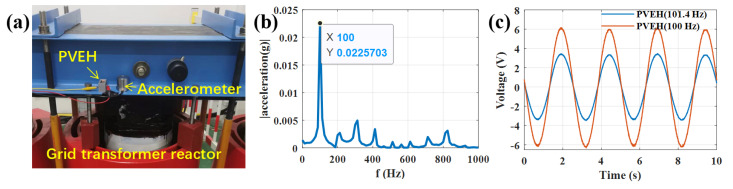
PVEH field application in grid transformer reactor: (**a**) Photo of PVEH field application; (**b**) Acceleration spectrum of grid transformer reactor; (**c**) Voltage time response curve at open circuit (load resistance of 10 MΩ).

To verify the actual effect of the PVEH, the 100 Hz PVEH and accelerometer are mounted on the tank of a grid transformer reactor (400 V 100 kVA), as shown in Figure 7. Acceleration spectrum (through Fourier transform) shows the main resonant frequency and amplitude are 100 Hz and 0.0226 g, respectively. Our PVEH with added mass has open circuit resonant frequency of 101.4 Hz. If we do not adjust the PVEH resonant frequency, the PVEH open-circuit voltage in 100 Hz is 3.6 V. When we adjust the PVEH resonant frequency to 100 Hz by magnetic frequency modulation, the open-circuit voltage is 6.1 V. Therefore, employing our tunable PVEH can handle the resonant frequency mismatch and obtain more efficient energy harvesting.

## 4. Conclusions

Based on the finite element simulation of double L-shaped mass piezoelectric cantilever beam structure, this paper presents the PVEH structure parameter optimization design method of 100 Hz target resonant frequency. It is considered that the peak power can be obtained as long as the short-circuit and open-circuit resonant frequency range includes 100 Hz. The PVEH aluminum alloy frame by 3D printing is integrated and free of bolt assembly, and the gluing packaging process has higher mechanical quality factor than bolt clamping. The experimental results of the prototype show that the peak power is 294 μW under the excitation of 0.1 g acceleration at 100 Hz and power density up to 5.28 μW mm^−3^ g^−2^. Experimental results show load resistance adjustment can achieve broadband peak power from 98.8 Hz (short circuit state) to 106.3 Hz (open circuit state). By adding mass of 3.8 g, resonant frequency of PVEH prototype can be reduced 4.9 Hz. In the magnetic coupling frequency modulation test, transverse force has more frequency modulation ability than axial force, with a frequency modulation band of 7.7 Hz and 4.1 Hz, respectively. Finally, tunable PVEH field application in grid transformer reactor is proven efficient at energy harvesting.

## Figures and Tables

**Table 1:** PVEH design parameters.

*Symbol*	*Parameters*	*Value*
*l_c_*	Cantilever length	28 mm
*w_c_*	Cantilever width	14 mm
*t_p_*	Piezoelectric layer thickness	0.4 mm
*l_p_*	Piezoelectric layer length	20mm
*t_s_*	Substrate layer thickness	0.2 mm
*t_bm_*	Big mass thickness	5 mm
*t_sm_*	Small mass thickness	2 mm
*l_bm_*	Big mass length	22 mm
*l_sm_*	Small mass length	10 mm
*ρ_m_*	Tungsten density	17,800 kg/m^3^
*ρ_p_*	PZT density	7500 kg/m^3^
*D_mag_*	Magnet diameter	4 mm
*t_mag_*	Magnet thickness	2 mm

**Table 2:** PVEH system parameter identification results.

Symbols	Parameters	Values
*C_p_*	Clamped capacitance	8.35 nF
*K*	System stiffness	10,298 N/m
*f_sc_*	Short circuit resonant frequency	98.8 Hz
*f_oc_*	Open circuit resonant frequency	106.3 Hz
*θ*	Electromechanical coupling factor	0.0037 N/V
*n_s_*	Structural loss factor	0.04218
*M*	Equivalent mass	26.723 g
*A*	Acceleration	0.1 g
*Q*	Mechanical quality factor	23.708
*k_e_^2^*	Effective electromechanical coupling coefficient	0.1576
*FoM*	Figure of merit	3.7342

**Table 3:** The performance comparison of various PVEHs.

References	Material	Acc (g)	Freq (Hz)	Power (μW)	Active Volume (mm^3^)	NPD (μW mm^−3^ g^−2^)	Mechanical Quality Factor
Kim 2010 [25]	PZT-5A	0.25	109.5	530	1780	4.76	27.5
Janphuang 2014 [26]	PZT-5H	1	96	82.4	47.82	1.72	32
Tang 2016 [27]	PZT	3	101	321	37.3	0.96	12.5
Quintero 2014 [28]	PZT-5H	0.1	49.8	6.7	57.3	11.7	42
Wang 2019 [10]	PZT-5H	1	160	2490	880	2.83	7.3
This work	PZT-5H	0.1	100	294	5566	5.28	23.7

## Data Availability

The data that support the findings of this study are available from the corresponding author upon reasonable request.
